# Management of Sepsis and Septic Shock: What Have We Learned in the Last Two Decades?

**DOI:** 10.3390/microorganisms11092231

**Published:** 2023-09-04

**Authors:** Shiwani Kamath, Hiba Hammad Altaq, Tony Abdo

**Affiliations:** Section of Pulmonary, Critical Care and Sleep Medicine, The University of Oklahoma Health Sciences Center, The Oklahoma City VA Health Care System, Oklahoma City, OK 73104, USA; shiwani-kamath@ouhsc.edu (S.K.); hiba-hammadaltaq@ouhsc.edu (H.H.A.)

**Keywords:** sepsis, septic shock, vasopressors, early goal-directed therapy, surviving sepsis campaign, crystalloids, colloids, corticosteroids

## Abstract

Sepsis is a clinical syndrome encompassing physiologic and biological abnormalities caused by a dysregulated host response to infection. Sepsis progression into septic shock is associated with a dramatic increase in mortality, hence the importance of early identification and treatment. Over the last two decades, the definition of sepsis has evolved to improve early sepsis recognition and screening, standardize the terms used to describe sepsis and highlight its association with organ dysfunction and higher mortality. The early 2000s witnessed the birth of early goal-directed therapy (EGDT), which showed a dramatic reduction in mortality leading to its wide adoption, and the surviving sepsis campaign (SSC), which has been instrumental in developing and updating sepsis guidelines over the last 20 years. Outside of early fluid resuscitation and antibiotic therapy, sepsis management has transitioned to a less aggressive approach over the last few years, shying away from routine mixed venous oxygen saturation and central venous pressure monitoring and excessive fluids resuscitation, inotropes use, and red blood cell transfusions. Peripheral vasopressor use was deemed safe and is rising, and resuscitation with balanced crystalloids and a restrictive fluid strategy was explored. This review will address some of sepsis management’s most important yet controversial components and summarize the available evidence from the last two decades.

## 1. Introduction and Historical Perspective

The word sepsis comes from the Greek word “σήψις,” meaning decomposition or decay. Its first documented use was over 2700 years ago when it appeared in Homer’s poems and was later utilized in the work of Hippocrates and Galen [1]. When the “Germ theory” of disease was developed in the 1800s, there was some recognition that sepsis is caused by dangerous micro-organisms. Hugo Schottmüller used the word sepsis in 1914 in the modern sense, writing that “sepsis is present if a focus has developed from which pathogenic bacteria, constantly or periodically, invade the bloodstream in such a way that this causes subjective and objective symptoms” [2].

The last two to three decades have seen continuous and increased efforts to better understand the pathophysiology of sepsis, refine its definition, and improve its management. In 1991, at the American College of Chest Physicians/Society of Critical Care Medicine Consensus Conference (ACCP/SCCM), the work of Roger Bone and his colleagues helped pave the way to the first consensus on the definition of sepsis. Sepsis was defined as a systemic response to infection, manifested by two or more of the systemic inflammatory response syndrome (SIRS) criteria. Severe sepsis was defined as sepsis associated with organ dysfunction, hypoperfusion, or hypotension, and septic shock as sepsis-induced hypotension that persists despite adequate fluid resuscitation [3]. In 2001, an international task force expanded the diagnostic criteria associated with sepsis, but despite the major limitations of the 1991 definitions, no major revision in definitions occurred given the lack of supporting evidence [4]. In 2016, the third international consensus definitions for sepsis and septic shock (sepsis-3) defined sepsis as a life-threatening organ dysfunction caused by a dysregulated host response to infection, and the term severe sepsis was aborted. The new definition incorporated the use of an acute change in the Sequential Organ Failure Assessment (SOFA) Score of ≥2 points to identify organ dysfunction. Septic shock was defined as a subset of sepsis with persisting hypotension, requiring vasopressor therapy to elevate MAP ≥ 65 mm Hg, and having serum lactate > 2 mmol/L despite adequate fluid resuscitation (Table 1) [5].

The surviving sepsis campaign, developed by the SCCM, the European Society of Intensive Care Medicine (ESCIM), and the International Sepsis Forum, was launched during the 2002 annual ESCIM meeting. Subsequent work led to the development and publication of the first guidelines for the management of sepsis and septic shock in 2004. These guidelines have been revised in 2008, 2012, 2016, and 2021 [6].

This article will mainly review the evolution of sepsis and septic shock management, focusing on initial resuscitation and early goal-directed therapy, intravenous fluid choices, vasopressor preferences, and corticosteroid controversies.

## 2. Epidemiology

The true epidemiology of sepsis continues to be highly debatable. The evolution of definition, screening, and management over the years, in addition to changes in coding and billing practices, increases the risk of sepsis being over- or underreported. Global assessments are even more difficult to obtain, as standardized coding of diagnoses is less prevalent internationally. In their assessment of global incidence and mortality of hospital-treated sepsis, Fleischmann et al. found that epidemiological data for low- and middle-income countries are almost nonexistent. In this meta-analysis, primarily based on data from high-income countries, sepsis incidence was 288 per 100,000 person-years, and hospital mortality was 17–26% [7]. A 2020 study published in the Lancet looking into global, regional, and national sepsis incidence and mortality between 1990 and 2017 showed a decline in age-standardized sepsis incidence and mortality consistent with prior studies, but the overall worldwide incidence was at least twice than thought previously [7,8,9]. This significant increase in worldwide incidence was mainly driven by the inclusion of data from lower socio-demographic areas, where sepsis incidence in some areas is as high as 4000 per 100,000 person-years and is responsible for >50% of total deaths [8]. Sepsis is likely responsible for more deaths worldwide than any other disease, and its impact on low- and middle-income countries is the highest. Global efforts are needed to tackle this crisis, obtain more epidemiological data, and design studies specific to this population to whom current management guidelines may not apply [6,10,11].

## 3. Pathophysiology

Prior to the 1997 work of Bone et al., excessive inflammatory response was thought to be the etiology of sepsis-like clinical manifestations. However, his work pioneered the idea that the initial inflammatory response in sepsis was also associated with a subsequent compensatory anti-inflammatory response [12]. Further research supports the idea that both inflammatory and anti-inflammatory responses occur and are more prolonged and complex than previously thought. While these responses may lead to tissue recovery and eradication of the causal pathogens, they can also cause organ dysfunction and secondary infections due to immunosuppression. Various factors dictate a patient’s clinical course, including age, co-morbidities, genetic predisposition, and the viral load and virulence of the pathogen itself [13].

The inflammatory and anti-inflammatory response’s net effect is unpredictable and highly individualized, leading to diagnostic and management challenges. Despite the increase in understanding complex immunological interactions, pro- and anti-inflammatory pathways, and pro- and anti-coagulation cascades in sepsis, it did not reflect in an improvement in management, as neither inflammatory markers removal nor targeted immunotherapy has shown a significant benefit [14]. An attempt to modulate the inflammatory, procoagulant, and fibrinolytic host response using recombinant human-activated protein C (Drotrecogin alfa, activated) in severe sepsis had promising results, with a 2001 study showing a reduction in mortality [15]. However, this was refuted in 2012 with a randomized controlled trial showing no reduction in 28- or 90-day mortality and an increase in serious bleeding risk, after which the product was withdrawn from the market [16].

A detailed review of sepsis pathophysiology is beyond the scope of this manuscript. Nevertheless, we wanted to highlight the complexity of pathways responsible for sepsis and the difficulty of optimally regulating inflammatory, immune, and coagulable responses to improve outcomes.

## 4. Screening for Sepsis

Over the years, multiple clinical variables and tools have been suggested to screen for sepsis or predict sepsis-related hospital mortality, and none were found to be perfect. In 2016, qSOFA (quick SOFA), a new simple model, was developed by Seymour et al. and incorporated into the sepsis-3 definition guidelines as a predictor for in-hospital mortality and prolonged ICU stay. It included altered mentation, systolic blood pressure ≤100 mm Hg, and respiratory rate ≥22/min (1 point each; score range, 0–3). Patients with infection and qSOFA ≥ 2 were designated as more likely to have poor outcomes [5,17]. Outside of the ICU, qSOFA performed very well with a predictive validity similar to SOFA and superior to SIRS. This was not the case in ICU patients where a change in SOFA score of ≥2 had greater prognostic accuracy for in-hospital mortality than qSOFA [17,18]. In an international prospective cohort study aimed to validate qSOFA in patients presenting to the emergency department with suspected infection, qSOFA had greater prognostic accuracy for in-hospital mortality than either SIRS or severe sepsis [19]. In a large meta-analysis (10 studies, *n* = 229,480) comparing SIRS and qSOFA, SIRS had a higher sensitivity and was superior to qSOFA for sepsis diagnosis, and qSOFA had a higher specificity and was better than SIRS in predicting hospital mortality [20]. Although qSOFA was not designed to be a screening tool for sepsis, uncertainty regarding its optimal use continued over the last few years, with several studies and critics emphasizing its inadequacy for sepsis screening and lower sensitivity compared to other tools like SIRS, MEWS (modified early warning score), and NEWS (National Early Warning Score) [21,22,23,24]. In 2021, the surviving sepsis guidelines recommended against using qSOFA compared to SIRS, NEWS, or MEWS as a single screening tool for sepsis and septic shock [6]. Screening tools alone, including automated EHR-based tools, do not have a mortality benefit. Nonetheless, they help in the early identification and treatment of sepsis and should be part of hospitals’ sepsis performance improvement programs, which are associated with a reduction in mortality [6,25].

## 5. Management

### 5.1. Initial Resuscitation: The Era of Early Goal-Directed Therapy and the Years After

In 1995, a multicenter randomized trial by Gattinoni et al. looking into goal-oriented hemodynamic therapy in critically ill patients failed to show a reduction in mortality using intravenous fluid resuscitation, blood transfusion, and inotropic agents to aim for supranormal cardiac index or normal mixed venous oxygen saturation [26]. In a landmark trial published in 2001, Rivers et al. hypothesized that a benefit might be seen if these interventions were implemented earlier and resuscitation endpoints were achieved sooner in patients with severe sepsis and septic shock. Patients randomized to receive six hours of early goal-directed therapy (EGDT) in the emergency department had a significantly lower mortality compared to the standard therapy group (30.5% vs. 46.5%; *p* = 0.009) [27]. This striking reduction in mortality led to the quick adoption of EGDT as the standard of care and its incorporation into the surviving sepsis campaign guidelines in 2004 [28]. However, several of these interventions and resuscitation endpoints did not stand the test of time and have been challenged over the years, showing no benefit, increased use of healthcare resources, and even potential harm. Between 2014 and 2015, three large multicenter randomized controlled trials, ProCESS (USA) [29], ARISE (Australia and New Zeeland) [30], and ProMISe (UK) [31], showed that hemodynamic management according to EGDT protocol did not lead to an improvement in outcome compared to usual care. This was also confirmed in PRISM, a meta-analysis of individual patient data of the three multicenter trials (ProCESS, ARISE, and ProMISe) published in 2017 [32]. EGDT and SSC efforts had likely contributed to prioritizing identifying and treating sepsis patients in the emergency department and improving “usual care.” However, EGDT as a protocolized care targeting CVP and ScVO2 goals in all patients with septic shock has no benefit, and subsequent SSC guidelines reflected that [6,33].

Based on Rivers et al.’s work, a quality measure called “Severe Sepsis and Septic Shock: Management Bundle” was developed in 2008, incorporated into the SSC guidelines, and endorsed by the National Quality Forum (NQF) [34]. In the 2012 SSC guidelines, the bundle was revised and split into four items that need to be completed within 3 h (measure lactate level, obtain blood culture before antibiotics, administer broad-spectrum antibiotics, and administer 30 mL/kg for hypotension or lactate ≥ 4 mmol/L) and three items within 6 h (apply vasopressors for hypotension that does not respond to initial fluid resuscitation to maintain a MAP ≥ 65 mmHg, measure CVP and ScvVO2 if persistent arterial hypotension post-fluid resuscitation or initial lactate ≥ 4 mmol/L, remeasure lactate if initial lactate was elevated) [35]. In 2013, the New York State Department of Health became the first state department of health to adopt the SSC bundle, and 10 years later, it remains the only one. In August 2014, the Centers for Medicare and Medicaid Services (CMS) adopted the Early Management Bundle for Severe Sepsis/Septic Shock (SEP-1) starting in October 2015, but removed the invasive monitoring requirement from EGDT in its final version, given the results of ProCESS, ARISE, and ProMISE trials that were published around the same time [29,30,31]. SEP-1 adoption by CMS divided healthcare providers into two camps, one in support and one opposing or at least questioning it given the conflicting evidence for benefit [36,37]. Reviewing data reported to the New York State Department of Health between 2014 and 2016, Seymour et al. showed that the delay in completion of the 3-h bundle was associated with higher in-hospital mortality in patients with sepsis and septic shock. This increase in mortality was driven by a delay in the administration of antibiotics and not a delay in fluid resuscitation [38]. In a 2018 editorial intended to be an update of the SSC bundle, Levy et al. proposed using an “hour-1 bundle” to replace the 3-h and 6-h bundles [39]. In the absence of strong (or even moderate) evidence for benefit, the adoption of a 1-h bundle carries the risk of unnecessary large volume fluid resuscitation and broad-spectrum antibiotics administration and may cause a significant burden on emergency departments that may potentially affect non- sepsis patients due to resources diversion [40]. Despite the New York State sepsis regulations leading to a reduction in sepsis mortality, it is unclear if the adoption of similar mandates in other states will have the same effect [41]. The reduction in sepsis-related mortality in New York could have been related to a relatively poor-quality sepsis care pre-mandate implementation and an extensive education campaign, not only SEP-1 compliance. In a large retrospective cohort published in 2021, including 117,510 patients from geographically diverse US hospitals using Cerner electronic health records, SEP-1 implementation was not associated with improved sepsis outcomes [42]. On the other hand, in a study published in 2022, Townsend et al. matched 112,870 Medicare patients whose care was compliant with SEP-1 with the same number of patients whose care was non-compliant and showed that compliance to SEP-1 reduced 30-day mortality by almost 5% [43]. The SEP-1 debate and the advocacy to keep it or remove it continues. The last SSC guidelines published in 2021 downgraded the recommendation for at least 30 cc/kg of intravenous crystalloid to be given in the first 3 h to a suggestion (weak recommendation) and allowed for further time (3 h instead of 1 h) to administer antibiotics in absence of shock and when sepsis is only “possible” [6].

### 5.2. Intravenous Fluids: Is There an Ideal Fluid, and How Much to Give?

Early intravenous fluid resuscitation to treat intravascular hypovolemia and restore adequate perfusion is a cornerstone of sepsis and septic shock management. Patients who present with sepsis will often receive large volumes of intravenous fluids, are at risk of acute kidney injury (AKI) and metabolic acidosis on presentation, may require renal replacement therapy, and are at higher risk of death [44]. Initial trials looked into using crystalloids versus colloids for resuscitation, and then interest shifted to compare balanced versus non-balanced crystalloids and conservative versus liberal fluid resuscitation strategies, with the ideal type and volume of intravenous fluid still up for debate.

#### 5.2.1. Colloids versus Crystalloids

Historically, colloids were used more often for resuscitation than crystalloids, and hydroxyethyl starch (HES) was the most commonly used. Data as early as 2001 suggested that HES is associated with acute kidney injury in patients with sepsis. Still, HES continued to be used under the presumption that newer HES with lower molecular weights (130/0.38–0.45 compared to 200/0.5–0.6) were safer [45,46]. In 2012, a large randomized controlled trial (Scandinavian Starch for Severe Sepsis/Septic Shock (6S)) comparing HES 130/0.42 to a lactate ringer showed that patients with severe sepsis resuscitated with HES were at higher risk of death at day 90 and more likely to receive renal replacement therapy [47]. The Crystalloid versus Hydroxyethyl Starch (CHEST) trial was also published the same year, showing no difference in mortality but a higher rate for renal replacement therapy in the HES group compared to saline [48]. Systematic reviews and meta-analyses followed and showed an increased risk for adverse events, blood transfusions, acute renal failure, and a potential increase in mortality with HES compared to crystalloids or albumin, marking the end of the HES era in sepsis [35,46,49]. An additional trial, the CRISTAL (Colloids Versus Crystalloids for the Resuscitation of the Critically Ill), was published in 2013 and found no difference in 28-day mortality between a mixed group of colloids (gelatins, dextrans, hydroxyethyl starches, or 4% or 20% of albumin) and crystalloids in patients with hypovolemic and septic shock. However, in this study, colloids demonstrated a benefit in mechanical ventilation duration, vasopressor use, and 90-day mortality (secondary outcomes), with no increase in renal replacement therapy. Yet, the many limitations of this study, including the 9-year recruitment period, the heterogeneity of the type of fluids received, and conflicting results with CHEST and 6S trials, make these benefits very questionable [50].

Albumin, a non-synthetic colloid, has been used over the years, with initial studies suggesting potential advantages over crystalloids in patients with severe sepsis. The Saline versus Albumin Fluid Evaluation (SAFE) trial, a large multicenter randomized controlled trial published in 2004, comparing 4% albumin and saline for fluid resuscitation in the intensive care unit, showed no difference in mortality, ICU length of stay, renal replacement therapy, and duration of mechanical ventilation. However, a prespecified subgroup analysis of 28-day mortality showed a non-significant trend favoring albumin in severe sepsis and normal saline in trauma, with a post hoc analysis in the trauma subgroup suggesting higher mortality with albumin in patients with traumatic brain injury [51]. In addition, a small single-center randomized controlled trial published in 2006 showed a potential benefit of albumin administration in critically ill patients with hypoalbuminemia [52]. In 2014, the Albumin Italian Outcome Sepsis (ALBIOS) study, a large multicenter open-label randomized controlled trial, showed that in patients with severe sepsis, resuscitation with 20% albumin and crystalloids did not improve survival compared to crystalloids alone. Albumin replacement in this study was associated with slightly better hemodynamics, reduced duration of vasopressor or inotropic support, and a lower cumulative net fluid balance. In addition, a post hoc subgroup analysis of septic shock patients showed a lower 90-day mortality in the albumin group, which was not the case for sepsis [53]. Nevertheless, the relevance of this finding is debatable, given it was a post hoc analysis of a secondary outcome that was not prespecified. A systemic review and meta-analysis also suggest that resuscitation with albumin may be associated with reduced mortality [49]. In the absence of harm, and potential benefit in some subgroups, the 2021 SSC guidelines included a weak recommendation suggesting using albumin in patients who received large volumes of crystalloids [6]. The ARISS (Albumin Replacement in Septic Shock) trial, a multicenter randomized controlled trial designed to investigate albumin replacement in septic shock given the potential benefit in prior studies, is actively recruiting and may provide additional future answers [54].

**Figure 1:**
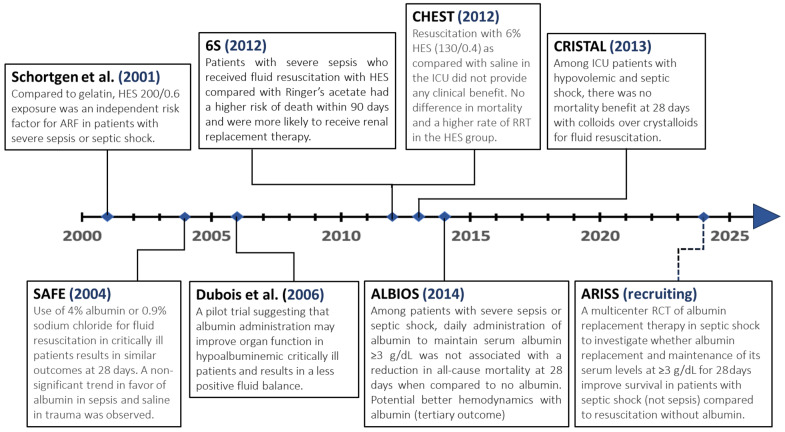
Key randomized controlled trials investigating resuscitation with different colloids and crystalloids in critically ill patients, including patients with sepsis and septic shock. HES: hydroxyethyl starch, ARF: acute renal failure, RRT: renal replacement therapy, ICU: intensive care unit.

Below is a 2-decade timeline summarizing key randomized controlled trials comparing resuscitation with colloids and crystalloids in critically ill patients (Figure 1).

#### 5.2.2. Balanced Crystalloids versus Saline

When managing patients with sepsis, clinicians often face the decision of choosing between balanced solutions (such as Plasma-Lyte and Lactated Ringers) or non-balanced solutions, such as 0.9% normal saline. Historically, 0.9% normal saline has been the most accessible and extensively used solution in the United States [55]. However, over the last decade, 0.9% normal saline became a less popular solution in sepsis due to its hyperchloremic characteristics and association with metabolic acidosis and AKI. The high chloride concentration of 0.9% normal saline is thought to mediate vascular smooth muscle contraction and amplify norepinephrine and angiotensin II-induced vasoconstriction, resulting in reduced renal blood flood, decreased renal perfusion, and AKI [56,57]. The increased risks of acidosis, inflammatory response, hypotension, and death associated with the high chloride content of saline have been demonstrated in experimental rat models of severe sepsis [58,59]. In healthy humans, the infusion of two liters of 0.9% normal saline significantly reduced renal artery blood velocity, renal cortical tissue perfusion, urine production, and extravascular fluid buildup compared to Plasma-Lyte [60].

In 2012, a prospective, open-label, sequential period pilot trial (*n* = 1533), alternating a chloride restrictive and a chloride liberal intravenous fluid strategy in a single center intensive care unit, showed a significant decrease in AKI and RRT with the restrictive chloride strategy [56]. However, this benefit was not seen in the SPLIT trial, a multicenter, double-blind, cluster-randomized, double-crossover trial published in 2015, designed to investigate the effect of a balanced crystalloid on renal complications in patients admitted to the intensive care unit compared to Saline. The study enrolled around 1100 patients in each group and showed no significant difference in AKI at 90 days, with 9.6% of patients in the Plasma-Lyte group developing AKI compared to 9.2% in the saline group. In addition, there was no difference in the rate of RRT use or in-hospital mortality between the two groups. The modest volume of fluid infused (median 2 L) and the small percentage of critically ill and septic patients made many question the lack of benefit in sicker critically ill patients [61]. In 2017, the SALT (Saline against Lactated Ringer’s or Plasma-Lyte) trial, a pilot trial (*n* = 974) primarily designed to assess the use of software tools within the electronic health record to compare saline to balanced crystalloids in the intensive care unit, also did not show a reduction in a composite outcome including mortality, new RRT, or persistent renal dysfunction (Major Adverse Kidney Event within 30 days—MAKE30) with balanced crystalloids [62]. In 2018, two pragmatic, multiple-crossover trials conducted concurrently in a large university-affiliated hospital were published, with results favoring balanced crystalloids in non-critically ill (SALT-ED) and critically ill patients (SMART) [63,64]. The Isotonic Solutions and Major Adverse Renal Events (SMART) trial enrolled 15,802 patients across 5 intensive care units of a single academic center, with each ICU alternating between balanced crystalloids or normal saline every month. Patients received saline or balanced crystalloids depending on the randomization of the unit to which they were admitted. The study showed a significant reduction in the composite outcome in the group receiving balanced fluids (14.3%) compared to saline (15.4%). However, there was no significant difference in individual components of MAKE30. The difference in outcomes appeared to be mostly driven by medical ICU patients and patients with sepsis or a history of renal replacement therapy [65]. A secondary analysis of the SMART trial, including only patients admitted to the medical ICU with sepsis (*n* = 1641), also showed a benefit of using balanced crystalloids in this population, as it was associated with lower 30-day in-hospital mortality (26.3% vs. 31.2%) compared to saline. In 2020, a meta-analysis published by Hammond et al., including 13 randomized trials of critically ill adults resuscitated with balanced crystalloids or saline, showed that in the sepsis cohort, hospital mortality was similar but the odds for MAKE30 were less with balanced crystalloids [66]. Given the limitations of the SMART trial (single-center, unblinded, lack of individual patient randomization) and the overall low quality of cumulative evidence, the 2021 SSC guidelines only “suggested” (weak recommendation) using balanced crystalloids instead of normal saline for resuscitation pending further studies [6].

Since the publication of the 2021 SSC guidelines, two large multicenter randomized controlled trials (BaSICS, and PLUS) have been published, showing no significant difference in outcomes between balanced solutions and saline in critically ill patients [57,67]. The Balanced Solution Intensive Care Study (BaSICS), published in 2021, was a large multicenter, double-blind, randomized control trial conducted in 75 intensive care centers in Brazil. A total of 11,052 patients were randomized to Plasma-Lyte 148 or 0.9% normal saline for all ICU fluid administration, with 90-day survival as the primary outcome. The study failed to show a survival benefit, with a 90-day mortality of 26.4% in the balanced solution group compared to 27.2% in the saline group (adjusted HR, 0.97 (95% CI, 0.90–1.05)). There was also no difference in secondary outcomes including the rate of AKI, RRT, and the potential harm for traumatic brain injury patients with a balanced solution [67]. The Plasma-Lyte 148 versus Saline (PLUS) Study was another large double-blind, randomized, controlled trial conducted in 53 ICUs in Australia and New Zealand, with the primary outcome being death from any cause at 90 days. A total of 5037 patients were randomized to either Plasma-Lyte 148 or saline. No difference was seen in the primary outcome with 90-day all-cause mortality of 21.8% in the Plasma-Lyte 148 group compared to 22% in the saline group. No difference was seen in any of the secondary outcomes, including newly initiated RRT or maximum increased creatinine level.

**Figure 2:**
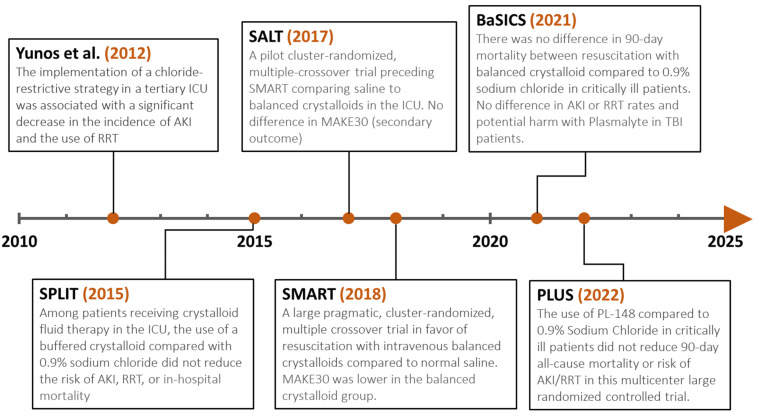
Key randomized controlled trials comparing balanced crystalloids to normal saline in critically ill patients, including patients with sepsis and septic shock. AKI: acute kidney injury. MAKE 30: Major Adverse Kidney Events within 30 days, a composite endpoint of mortality, treatment with kidney replacement therapy, and/or doubling creatinine, PL-148: Plasma-Lyte 148, RRT: renal replacement therapy, TBI: traumatic brain injury.

The findings of the BaSICS and PLUS trials, along with the results of the SPLIT trial, appear inconsistent with the SMART trial outcomes, making the 2021 SSC suggestion in favor of balanced crystalloids in sepsis patients even weaker. Following the PLUS trial, an updated meta-analysis by Hammond et al., including the results of BaSICS and PLUS, was published in 2022. This meta-analysis suggested a high probability for a decrease in mortality with balanced crystalloids, albeit not statistically significant, with the effect of using balanced crystalloids versus saline ranging from a 9% relative reduction to a 1% relative increase in the risk of death [68].In today’s practice, insufficient evidence exists to recommend balanced crystalloids over saline across a heterogenous ICU population. Patients’ characteristics, underlying comorbidities, and the availability and cost of balanced crystalloids may play a role in choosing the most suitable fluid. For example, a potential benefit for balanced crystalloid is seen in patients who require large amounts of fluid, patients with diabetes ketoacidosis, or with existing hyperchloremic metabolic acidosis [57,64,69,70]. On the other hand, potential harm exists in patients with traumatic brain injury [67]. More studies are needed to identify various subsets of patients who will benefit the most from a chloride-restrictive fluid resuscitation strategy (Figure 2).

#### 5.2.3. Liberal vs. Restrictive Fluid Resuscitation Strategies

Fluid resuscitation is regarded as a key therapeutic measure for the initial management of patients with sepsis or septic shock and is thought to improve clinical outcomes [28,34,35]. The lack of evidence regarding the amount of fluid needed to resuscitate septic patients appropriately led to significant variation in practice, with patients being either over-resuscitated, under-resuscitated, or resuscitated late after transfer to the intensive care unit. Rivers’ trial standardized fluid resuscitation by enrolling patients with sepsis with persistent hypotension after a crystalloid fluid challenge of 20 to 30 cc/kg over 30 min, and then a 500 mL bolus was given every 30 min to achieve a CVP of 8–12 mmHg. This strategy resulted in a significantly higher amount of fluid being administered in the first 6 h in the EGDT group compared to the standard of care (5 L vs. 3.5 L), but both groups ended up receiving the same amount of fluid (13.4 L) at 72 h. Given the significant reduction in mortality with the EGDT trial, the SSC adopted this protocolized quantitative resuscitation strategy until its 2016 update, when recommendations changed in light of three large multicenter randomized controlled trials (PROCESS, ARISE, and PROMISE showing no benefit with this approach. As the average volume of fluid pre-randomization was approximately 30 mL/kg in these three trials, the SSC guidelines “strongly” recommended at least 30 mL/kg of IV crystalloid fluid be given within the first 3 h, despite “low-quality evidence” of a benefit [29,30,31,33]. In addition, given emerging data showing that the static measurement of CVP poorly predicts fluid responsiveness, the SSC dropped CVP measurement as guidance for fluid resuscitation and suggested dynamic over static variables to predict fluid responsiveness (weak recommendation) [33,71,72]. The 30 mL/kg continued to be challenged given the low level of certainty for this evidence, with some data from small trials pointing toward the benefit of a restrictive fluid strategy. A systematic review and meta-analysis by Silversides et al. showed that in adults with sepsis or acute respiratory distress syndrome, a conservative or deresuscitative fluid strategy resulted in increased ventilator-free days and a decreased length of ICU stay compared to a liberal strategy or standard care without an increase in acute kidney injury or renal replacement therapy [73]. In low-income countries with limited resources, a liberal fluid strategy was associated with an increased risk of respiratory failure and death [74]. This outcome is likely explained by differences in patients’ characteristics, underlying diseases, and lack of sufficient mechanical ventilation support in these countries. In one study, early norepinephrine was associated with increased shock control at 6 h and a lower incidence of cardiogenic pulmonary edema in patients with septic shock [75]. This approach may be beneficial in settings lacking appropriate ventilator support. These populations-and resource-dependent findings raise many questions regarding the validity of a universal resuscitation approach in all sepsis patients [11,74,76]. In a multicenter retrospective analysis published in 2017, Seymour et al. showed no association between mortality and time to completion of fluid bolus during mandated emergency care for sepsis in New York [38]. In 2020, Meyhoff et al. published a systematic review involving 637 patients from 9 randomized controlled trials published after 2015, showing no difference between lower versus higher fluid volume groups in all-cause mortality or any of the secondary or exploratory outcomes. Nonetheless, the authors acknowledged the scarcity of evidence to support either approach [77].

The 2021 SSC guidelines acknowledged the lack of good data to recommend using a restrictive versus liberal fluid strategy after the initial fluid bolus is given. In 2022, the Conservative versus Liberal Approach to Fluid Therapy in Septic Shock (CLASSIC) trial was published, showing no difference in 90-day mortality or any secondary outcomes between restrictive and standard fluid therapy. This study included 1554 patients in septic shock from 31 European ICUs randomized to receive restricted intravenous fluid or standard intravenous fluid therapy. It is important to note that included patients had to receive at least 1 L of fluid before randomization. Based on this design, the study did not address initial fluid resuscitation and did not challenge the 30 cc/kg bolus [78]. More recently, the anticipated CLOVERS (Crystalloid Liberal or Vasopressors Early Resuscitation in Sepsis) trial was published. This study was conducted by the PETAL network and enrolled 1563 patients in 60 US centers randomized to either a restrictive strategy prioritizing early vasopressor use or a liberal strategy, with the hypothesis that all-cause mortality before discharge home by day 90 would be lower with a restrictive fluid strategy. The study showed no difference in the primary outcome (death before discharge home by day 90) or any secondary outcomes, including new invasive mechanical ventilation or RRT initiation. The difference in IV fluid administered in the first 24 h was around 2.1 L (1.2 L vs. 3.2 L). Although CLOVERS did not demonstrate the superiority of a restrictive fluid approach, it showed non-inferiority [79].

Future studies comparing restrictive and liberal fluid strategies should try to identify subgroups of patients who may benefit from one approach over the other. The current evidence supports that both approaches appear safe, empowering clinicians in 2023 to individualize fluid resuscitation after the first bolus based on their analyses of patients’ fluid status and responsiveness, pending the identification of more sophisticated methods that allow better recognition of those subgroups.

### 5.3. Vasopressors

#### 5.3.1. Choice of Vasopressor Agents

The choice of initial vasopressor has been evaluated over the years with several randomized controlled trials and meta-analyses, leading to norepinephrine being recommended as the first-line agent in septic shock. Yet, the quality of evidence differs for various vasopressors, ranging from high-quality evidence for dopamine, moderate quality for vasopressin, low for epinephrine and selepressin, and very low for angiotensin II. The 2021 SSC guidelines recommend using norepinephrine as the first-line vasopressor agent, with weak recommendations to add vasopressin next, followed by epinephrine [6]. This paragraph will discuss the available evidence and potential future research.

#### 5.3.2. Dopamine versus Norepinephrine

The first SSC guidelines published in 2004 recommended either norepinephrine or dopamine through a central venous catheter as the first-choice vasopressor agent to correct hypotension in septic shock [28]. However, a large 2010 landmark trial (SOAP II, *n* = 1679) comparing dopamine and norepinephrine in the treatment of shock showed no difference in 28-day mortality but a significantly higher risk of arrhythmia with dopamine (24.1% vs. 12.4%, *p* < 0.001), making norepinephrine the vasopressor of choice for septic patients in the 2012 SSC guidelines update [35,80]. Furthermore, in a 2015 systemic review and meta-analysis of 11 randomized controlled trials comparing norepinephrine and dopamine, norepinephrine had a better hemodynamic profile, lower risk for adverse events and arrhythmia, and overall lower mortality (RR 0.89; 95% CI 0.81–0.98) [81]. Hence, in 2023, dopamine use in septic shock should be limited to selective patients with bradycardia or if norepinephrine is unavailable.

#### 5.3.3. Vasopressin versus Norepinephrine

Patients with septic shock are thought to have relative vasopressin deficiency [82,83]. Early case series and small randomized studies showed a decrease in catecholamines’ requirement and a potential renal protective effect with vasopressin [84,85]. These findings prompted an increase in vasopressin use in the early 2000s, despite the absence of large randomized controlled trials showing benefits [83]. In 2008, a large randomized controlled trial (VASST, *n* = 778) showed no difference in 28-day mortality when vasopressin was added to norepinephrine compared to norepinephrine alone. However, in a subgroup analysis, a trend toward lower 28-day mortality (26.5% vs. 35.7%, *p* = 0.05) was observed with the addition of vasopressin to norepinephrine in patients with lower severity shock (norepinephrine dose < 15 µg/min) [86]. After VASST, several systemic reviews and meta-analyses had conflicting findings related to the potential survival benefit of vasopressin [87,88,89]. Further analysis from VASST also suggested that vasopressin may decrease the risk of kidney failure [90]. The VANISH (Vasopressin vs. Norepinephrine as Initial Therapy in Septic Shock) trial, published in 2016, directly compared the use of vasopressin to norepinephrine (instead of an add-on to norepinephrine as in VASST), with the primary outcome being 28-day survival free from kidney failure. The study showed no significant difference between vasopressin and norepinephrine in the number of kidney failure-free days or 28-day mortality (30.9% vs. 27.5%; RR 1.13 (95% CI 0.85–1.51)). Although vasopressin did not decrease the risk of kidney injury, its use was associated with a lower rate of renal replacement therapy (RRT). This potential benefit was mainly driven by the non-survivor group, so it needs to be interpreted cautiously [91]. Recent systemic reviews and meta-analyses show that vasopressin is safe in septic shock, reduces the risk for arrhythmia when combined with catecholamines, and may decrease the rate of RRT, but has no effect on mortality and may be associated with a higher risk for digital ischemia [92,93]. The 2021 SSC guidelines suggest starting vasopressin when the norepinephrine dose reaches 0.25–0.5 μg/kg/min [6]. The perfect timing to start vasopressin in septic shock continues to be debatable, with some in favor of initiating it early on with a lower dose of norepinephrine [94,95].

#### 5.3.4. Epinephrine versus Norepinephrine

Over the last 20 years, norepinephrine has always been preferred over epinephrine as a first-line vasopressor agent in septic shock. The preference toward norepinephrine is primarily driven by an overall higher risk for adverse events seen with epinephrine [6]. Efficacy-wise, one randomized controlled trial failed to show a difference between norepinephrine and epinephrine [96]. Another multicenter randomized controlled trial (*n* = 330) comparing epinephrine alone to norepinephrine plus dobutamine in septic shock also showed no significant difference in efficacy and mortality [97]. Epinephrine has been associated with an increased risk of splanchnic vasoconstriction, hyperlactatemia, hyperglycemia, and tachyarrhythmias [96,97,98]. The optimal timing to add epinephrine to norepinephrine in patients with septic shock is unclear and may need to be individualized [6,94]. At present, epinephrine may be considered a first-line agent in settings where norepinephrine is unavailable and in patients with severe septic shock and cardiac dysfunction [6].

#### 5.3.5. Phenylephrine versus Norepinephrine

Phenylephrine is a pure alpha agonist with pure arterial and venous vasoconstriction, and its effect on cardiac output is variable. It is a significantly less potent vasopressor than norepinephrine, and phenylephrine monotherapy is unlikely to be enough in patients with septic shock. A small randomized controlled trial (*n* = 32) compared phenylephrine to norepinephrine for initial hemodynamic support of patients with septic shock showed no significant differences. However, patients in the norepinephrine group achieved a higher mean arterial pressure at a significantly lower vasopressor dose compared to phenylephrine, and half of the patients in each group also received dobutamine, making it hard to conclude, based on this small study, that phenylephrine monotherapy is equivalent to norepinephrine [99]. In addition, a large retrospective cohort (*n* = 27,835) that assessed changes to patient care and outcomes associated with the 2011 national norepinephrine shortage showed a higher in-hospital mortality during the shortage, with phenylephrine being the most commonly administered alternative vasopressor [100]. Despite being overall inferior to norepinephrine, phenylephrine still appeals to some intensivists in patients with septic shock and atrial fibrillation, given that it is associated with lower heart rate than norepinephrine [101]. Yet, whether or not these modest reductions in heart rate are associated with meaningful clinical outcomes requires further study.

#### 5.3.6. Angiotensin II versus Norepinephrine

Angiotensin II causes vasoconstriction through stimulation of the renin-angiotensin system. Its synthetic form became recently available and was approved by the FDA in 2017 for septic or other distributive shock based on the result of the ATHOS-3 trial [102,103]. This trial included 344 patients randomized to angiotensin II or a placebo in addition to background vasopressors, with the primary endpoint being an increase in mean arterial pressure of at least 10 mmHg or at least 75 mmHg. The primary endpoint was achieved in 70% of the angiotensin II group compared to 23% in the placebo. The study showed no difference in mortality or any other patient-centered outcomes. There was no clear increase in adverse events with the use of angiotensin II, although the FDA reported an increase in thrombotic events [103]. Over the last five years, a couple of post hoc analyses of the ATHOS-3 trial, in addition to retrospective studies, showed a potential benefit to start angiotensin II before the norepinephrine dose exceeds 0.25 mcg/Kg/min and a signal for improved mortality and reduced renal replacement therapy [104,105,106,107]. These exploratory outcomes need to be verified with randomized controlled trials. In summary, until further evidence, angiotensin II should not be used as a first-line agent but may have some role as an adjunctive therapy [6]. Additional research is warranted concerning the timing and sequence of angiotensin II administration and identifying potential subgroups that benefit the most from it.

#### 5.3.7. Selepressin versus Norepinephrine

Selepressin is a selective vasopressin V1a receptor agonist, potentially mitigating sepsis-induced vasodilatation and vascular leakage. In a phase 2a trial in patients with septic shock, selepressin reduced norepinephrine requirement and was associated with a reduction in mechanical ventilation days [108]. These findings led to the SEPSIS-ACT, a multicenter randomized clinical trial that enrolled 828 patients with septic shock on norepinephrine to add on selepressin or the placebo. The trial, published in 2019, was stopped for futility as it failed to show any difference in the ventilator- and vasopressor-free days (primary endpoint) [109]. The 2021 SSC guidelines issued a weak recommendation against using selepressin as a first-line therapy [6]. Regardless, selepressin does not have FDA approval.

In 2023, the bulk of evidence still supports norepinephrine as the first-line vasopressor agent in septic shock. However, further research is needed to identify the best timing to add vasopressin as a second agent and the subgroups that would benefit most from adding angiotensin II before epinephrine.

#### 5.3.8. Central vs. Peripheral Administration

Intravenous vasopressors are used to manage patients with distributive shock with persistent hemodynamic instability after a fluid challenge. Historically, vasopressors have been delivered via a central venous catheter due to concerns of localized tissue injury from extravasation. Over the last 10 years, several small studies and systematic reviews showed that overall extravasation risk is <5% with no long-term sequela secondary to that. The risk may be higher when the vasopressors are infused with catheters distal to the antecubital fossa and for >6 h [110,111,112,113]. Data from the ARISE trial showed that 42% of patients had vasopressors initiated through peripheral lines with a shorter time for vasopressors’ initiation and no significant adverse outcomes [30]. Establishing the safety of vasopressors’ peripheral administration is of substantial importance for settings that lack the availability and expertise in central venous catheter insertion. A survey on critical care resources in Africa reveals that a central venous catheter is “never” available per 25% of the surveyed individuals [75]. A small pilot trial in Uganda showed that peripheral norepinephrine administration in septic shock patients outside the intensive care unit is feasible, safe, and may improve mortality in a low-resource setting [114]. The 2021 SSC guidelines issued a weak recommendation suggesting vasopressor initiation through a peripheral catheter until a central venous catheter is secured [6]. In 2023, the CLOVERS trial also showed that initial peripheral administration of vasopressor agents was safe, with only 3 extravasations among 500 patients who received vasopressors peripherally [79].

### 5.4. Anti-Infective Therapy

Antibiotic choice and timing remain integral components of the sepsis bundle. Over the last 20 years, attempts have been made to balance the benefit of early administration of appropriate antibiotherapy and the risks associated with treatment delay and unnecessary “wide spectrum” antibiotherapy.

#### 5.4.1. Antibiotic Timing

The 2021 SSC guidelines recommend the administration of antibiotherapy as soon as possible, ideally within 1 h for patients with septic shock or a high likelihood of sepsis [6]. However, in the absence of shock and as the probability of sepsis gets lower, the guidelines recommend further workup, ideally within the first 3 h, before deciding if antibiotherapy is warranted [6]. In addition, the level of evidence for the administration of antibiotherapy within the first hour was downgraded to “low” for septic shock and “very low” for sepsis compared to “moderate” for both in the 2016 SSC guidelines [6,33]. This change in guidelines reflects several studies showing that reduction in mortality with early administration of antibiotherapy is mainly seen in septic shock patients [115,116,117,118]. Still, despite the low quality of evidence, the 2021 SSC guidelines maintained a “strong” recommendation to administer antibiotic therapy within 1 h in potential septic shock patients due to the overall high risk of death and the association of poor outcomes with delayed antibiotherapy in this population [6,38,117,118]. These recommendations are very similar for resource-limited countries, although limited data exist [119]. A prospective multicenter cohort published in 2022, including around 3000 patients, showed an increased mortality risk for every 1 h of antibiotic delay in patients with septic shock but not in patients without shock [120]. Another recent large retrospective cohort, including 74,000 patients, showed that delay in antibiotherapy in patients with sepsis was associated with an increased risk of progression to septic shock but no significant increase in mortality [121]. Overall, the current data support the 2021 SSC recommendations.

#### 5.4.2. Antibiotic Choice

Initiation of inappropriate antibiotherapy in septic shock has been shown to result in a significant increase in mortality [122]. The patient’s medical history, including medical comorbidities and potential immunocompromised status, presence of indwelling devices, history of prior infections with multidrug-resistant microorganisms, previous hospitalizations, antibiotic administration, and the site of current infection must be considered when choosing an appropriate empiric antibiotic regimen. Therefore, empiric antimicrobials with coverage against methicillin-resistant staph aureus (MRSA) are only recommended if the patients are at high risk for MRSA (recurrent wound infections, hemodialysis patients, recurrent hospitalizations, history of MRSA infections or colonization, etc.) [6]. Observational data show that earlier MRSA coverage led to better outcomes in patients with known MRSA infection but may be associated with increased adverse events and mortality in MRSA-negative patients [123,124,125,126]. In patients with pneumonia with subsequent negative cultures for MRSA or negative nares MRSA, stopping empiric MRSA coverage led to better outcomes [127,128]. For multidrug-resistant (MDR) gram-negative rods (GNR) coverage, the 2021 SSC guidelines only “suggest” double antibiotic coverage in patients at high risk for MDR microorganisms and suggest against it in patients whose MDR risk is low [6]. A large meta-analysis including 13 RTCs showed no difference in mortality or other significant outcomes between mono vs. combination antibiotic therapy in adult ICU patients with severe sepsis [129]. Hence, choosing the appropriate antibiotic for GNR coverage or deciding on the need for double coverage must consider patients’ history, local antibiogram, and community rates of MDR.

#### 5.4.3. Antifungal Coverage

The risk of death is high among patients with septic shock attributed to fungal infection [130,131]. Still, the data have not fully supported early empiric antifungal therapy, especially in nonneutropenic patients [132,133]. A multicenter RCT showed that empirical treatment with micafungin compared to a placebo among nonneutropenic critically ill patients with ICU-acquired sepsis failed to show a survival benefit [134]. Thus, the 2021 SSC guidelines only “suggest” antifungal coverage in patients at high risk for fungal infections compared to “recommend” in 2016. The decision to empirically treat and the choice of the antifungal agent depend on many factors, both patient and infection-related, with the highest potential benefit for empiric therapy in neutropenic and post-transplant patients [135].

#### 5.4.4. Antibiotic Duration and Role of Sepsis Biomarkers

Daily assessment for antibiotic de-escalation is recommended, and antibiotics should be tailored to cultures and microorganism sensitivities when available [136]. Multiple factors affect antibiotic duration, including the source of infection, the responsible microorganism, adequate source control or lack thereof, and the patient’s immune and clinical status [137]. Historically, longer antibiotic courses were preferred, but over the last decade, there has been an overall movement toward a shorter duration of antibiotherapy, with cumulative data supporting this practice in pneumonia, intraabdominal infections, and non-complicated gram-negative bacteremia [138,139,140]. Infectious disease specialists’ consultation has been linked to shorter time to antibiotic de-escalation and improved sepsis outcomes, especially in patients with MRSA or MDR GNR [141,142].

Biomarkers such as procalcitonin should not be used to decide whether to start antibiotherapy in patients with suspected sepsis and septic shock. However, they may be used along with clinical evaluation to discontinue antibiotics [6]. Procalcitonin is the most studied sepsis biomarker in critically and non-critically ill patients [143]. Randomized controlled trials did not find any significant benefit of using procalcitonin to start antibiotics in sepsis patients, and guidelines for community-acquired pneumonia recommend initiating antibiotherapy regardless of procalcitonin serum level given its highly variable reported sensitivity and the concern for increased mortality when antibiotics are withheld based on a low procalcitonin serum level [144,145,146,147]. On the other hand, procalcitonin use to guide antibiotic discontinuation has been deemed safe and is associated with a shorter duration of antibiotherapy and potentially improved mortality [148,149,150]. Procalcitonin testing availability and cost may be an obstacle for use in low- and middle-income countries (LMICs). Small cohorts have shown a probable benefit for procalcitonin and C-reactive protein (CRP) use in LMICs. However, additional studies are needed to define potential beneficial scenarios and cost-effectiveness before advocating for broad usage. [151]. Monocyte distribution width is a new biomarker for infection and has shown to be at least equivalent to procalcitonin and CRP, if not superior [152,153].

The next decade will likely witness the emergence of additional sepsis biomarkers and the potential combination of these biomarkers in one test that might help with antibiotics’ initiation, choice, and duration.

### 5.5. Adjunctive Glucocorticoid Therapy

Anecdotal data regarding the use of steroids in sepsis date back to the 1950s, with small randomized controlled trials published in the 1960s and 1970s showing contradicting outcomes [154,155]. In the 1980s, multicenter trials attempted to answer controversial questions regarding steroid use, dosage, and potential subgroups that would benefit the most (presumably gram-negative rods septicemia). In 1987, two multicenter trials that randomized patients to high-dose methylprednisolone (30 mg/Kg) and a placebo were published, showing that high-dose corticosteroids have no benefits in the treatment of severe sepsis and septic shock and may potentially increase mortality related to secondary infections [156,157]. Following these studies, the IDSA recommended against the routine use of steroids in severe sepsis and septic shock in 1992 [158]. Several meta-analyses in the 1990s showed no benefits for steroids in the spectrum of sepsis [159,160]. A decade later, the concept of relative adrenal insufficiency in septic shock patients and systemic inflammation-induced glucocorticoid receptor resistance prompted renewed interest in longer courses of low-dose corticosteroids [161,162,163]. In 2002, Annane et al. showed that a 7-day course of low-dose hydrocortisone (50 mg intravenous every 6 h) and fludrocortisone (50 mcg daily) decreased mortality (53% vs. 63%) and the median duration of vasopressor therapy in patients with septic shock and relative adrenal insufficiency compared to a placebo. The study enrolled 300 adult patients with septic shock after undergoing a short corticotropin test to identify patients with relative adrenal insufficiency [164]. Given the result of this study, the 2004 SSC guidelines issued a recommendation to use corticosteroids in patients with septic shock [28]. In 2008, the Corticosteroid Therapy of Septic Shock (CORTICUS) trial was published, showing that hydrocortisone did not decrease mortality in patients with septic shock, regardless of their response to corticotropin [165]. Compared to the Annane et al. trial, CORTICUS randomized patients (*n* = 499) to a longer hydrocortisone taper (11 days) without fludrocortisone versus the placebo, enrolled patients within 72 h (compared to within 8 h), included fewer sick patients, as per SAPS II, upon enrollment, and lowered mortality in the placebo group (32% compared to 61%) [164,165]. These differences could have been responsible for the difference in outcomes between the two studies. Similar to the Annane et al. trial, the shock reversal was quicker with hydrocortisone, but in CORTICUS, a higher incidence of secondary infections was seen in the hydrocortisone group [164,165]. Given the result of CORTICUS, the 2008 SSC guidelines suggested (compared to “recommended” in 2004) that intravenous hydrocortisone be given only to adult septic shock patients after blood pressure is identified to be poorly responsive to fluid resuscitation and vasopressor therapy and that the ACTH stimulation test not be used [34]. Several systemic reviews and meta-analyses followed, some showing improved mortality and others not, but most showing improved shock reversal with hydrocortisone [166,167,168,169]. The Hydrocortisone for Prevention of Septic Shock (HYPRESS) trial, published in 2016, was the first RCT to randomize patients with severe sepsis (*n* = 380) to hydrocortisone (continuous infusion of 200 mg/day × 5 days, then tapered until day 11) or a placebo. It showed no difference in progression to septic shock (primary outcome) or mortality (8.8% vs. 8.2%) [170].

The 2016 SSC guidelines included no significant change compared to 2008, with a suggestion against using intravenous hydrocortisone to treat septic shock patients if adequate fluid resuscitation and vasopressor therapy can restore hemodynamic stability [33]. The status quo was shaken up a little bit a decade after the 2008 SSC guidelines with the concurrent publication of ADRENAL and APROCCHSS in 2018, the two largest randomized controlled trials examining the effect of steroids in septic shock [171,172]. The Adjunctive Corticosteroid Treatment in Critically Ill Patients with Septic Shock (ADRENAL) randomized 3658 patients with septic shock requiring mechanical ventilation to 200 mg/day and a continuous infusion of hydrocortisone or a placebo for 7 days. The study showed that hydrocortisone infusion did not result in a statistically significant reduction in 90-day mortality compared to the placebo (27.9% vs. 28.8%). Patients in the hydrocortisone group had faster resolution of shock than those in the placebo (median duration; 3 vs. 4 days). Although patients in the hydrocortisone group had a shorter duration of the initial episode of mechanical ventilation, there was no significant difference in the number of days alive and free of mechanical ventilation [171]. On the other hand, The Activated Protein C and Corticosteroids for Human Septic Shock (APROCCHSS) trial was a multicenter randomized trial with a two-by-two factorial design that was developed to test the hypothesis that hydrocortisone-plus-fludrocortisone therapy or activated Drotrecogin alfa would improve the clinical outcomes of patients with septic shock. After Xigris withdrawal from the market in 2011, the trial continued with 2 parallel groups and randomized 1241 patients to a hydrocortisone dose of 50 mg IV every 6 h plus 50 mcg oral fludrocortisone, or a placebo, for 7 days. Contrary to ADRENAL, this trial showed that treatment with hydrocortisone-plus-fludrocortisone resulted in lower 90-day mortality compared to the placebo (43% vs. 49%). Secondary outcomes also favored the hydrocortisone-plus-fludrocortisone group, which had a lower 180-day mortality and a higher number of vasopressor-free days and organ-failure-free days but a similar number of ventilator-free days compared to the placebo. No difference in the rate of serious adverse events was seen [172]. It is unclear why APROCCHSS showed a reduction in mortality with corticosteroids and ADRENAL did not. Is it the fludrocortisone effect or the possibility that APROCCHSS enrolled much sicker patients? And did corticosteroids offer a mortality benefit only in extremely sick patients? A much smaller randomized controlled trial (COIITSS) from 2010 showed that the addition of oral fludrocortisone did not result in statistically significant improvement in mortality, although it is possible that the study was underpowered to detect such a difference [173]. A systematic review and meta-analysis including 37 randomized controlled trials, including APROCCHSS and ADRENAL (*n* = 9564), suggested that corticosteroids are associated with reduced 28-day mortality compared with a placebo in adults with sepsis [174]. A Cochrane meta-analysis by Annane et al., including 61 RCTs (*n* = 12,192), also showed that corticosteroids probably reduced 28-day and hospital mortality [174,175]. In contrast, another systematic review and meta-analysis including 22 RCTs (n = 7297) showed no difference in mortality but a reduction in the duration of shock, mechanical ventilation, and ICU length of stay with low-dose corticosteroids in septic shock patients [176].

Driven by the results of APROCCHS, and ADRENAL, the 2021 SSC guidelines included a weak recommendation in favor of corticosteroids in patients with ongoing shock following fluid resuscitation (200 mg/day of hydrocortisone given as 50 mg intravenously every 6 h or as a continuous infusion in patients on ≥0.25 mcg/kg/min norepinephrine or epinephrine at least 4 h after initiation) [6].

**Figure 3:**
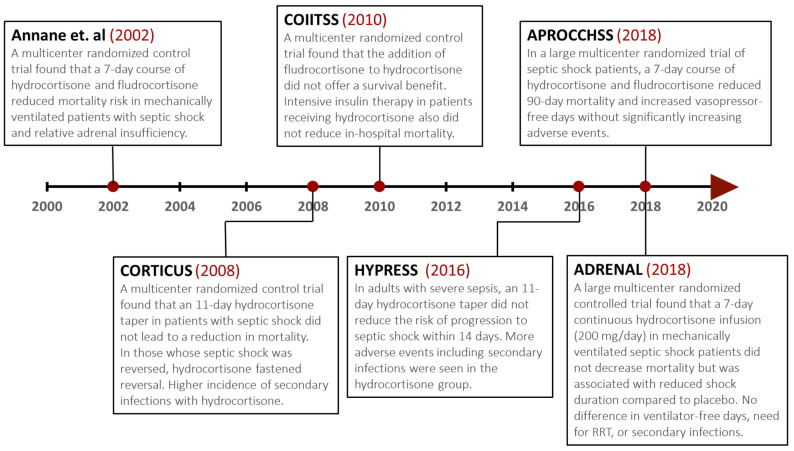
Key randomized controlled trials of corticosteroids in sepsis and septic shock. RRT: renal replacement therapy.

The debate regarding the association of low-dose corticosteroids with a mortality benefit in patients with septic shock and whether adding fludrocortisone has any additional benefit has yet to be settled. A recent multicenter cohort study including 88,275 patients showed that the addition of fludrocortisone was superior to hydrocortisone alone among adult patients with septic shock and was associated with a 3.7% lower adjusted absolute risk difference in the primary composite outcome of mortality or discharge to hospice [177]. In May 2023, a patient-level meta-analysis of low-dose hydrocortisone in adults with septic shock showed that hydrocortisone compared with a placebo was not associated with reduced 90-day mortality for patients with septic shock. There was also no significant difference in secondary outcomes, except in vasopressor-free days (mean difference, 1.24 days). However, this meta-analysis did not exclude the potential mortality benefit of hydrocortisone with fludrocortisone (RR of 0.86 for 90-day mortality) [178]. In our current practice, we use 200 mg/day of intravenous hydrocortisone plus 50 mg of oral fludrocortisone, given the potential benefit of fludrocortisone with minimal to no additional risks (Figure 3).

### 5.6. Administration of Red Blood Cells and Transfusion Threshold

In 1999, the TRICC (Transfusion Requirements in Critical Care) trial suggested that a restrictive red cell transfusion strategy (hemoglobin goal of 7–9 g/dL) is as effective and possibly superior to a liberal strategy (hemoglobin goal of 10–12 g/dL) in most critically ill patients, including patients with sepsis [179]. Based on this study, the 2004, 2008, and 2012 SSC guidelines recommended red blood cell transfusion when hemoglobin decreases to <7g/dL. However, this threshold excluded the early resuscitation phase recommending a hematocrit transfusion threshold of <30% in patients with mixed/central venous oxygen saturation <70% per EGDT [28,34,35]. In 2014, the TRISS trial, a large multicenter study, randomized 1005 patients with septic shock to a lower hemoglobin threshold (≤7 g/dL) for blood cell transfusion versus a higher hemoglobin threshold (≤9g/dL) and showed similar 90-day mortality, ischemic events, and use of life support with fewer transfusions in the lower threshold group [180]. In 2016, the SSC guidelines included a strong recommendation favoring a restrictive strategy (transfusion threshold < 7 g/dL) given the TRISS results and PROCESS, ARISE, and PROMISE showing no difference in mortality between EGDT and usual care [33]. In 2017, the TRICOP (Transfusion Requirements in Critically Ill Oncologic Patients), a small randomized single-center trial, showed a survival benefit with a liberal strategy (<9 g/dL) in adult cancer patients with septic shock. Yet, this result was considered to be only hypothesis-generating by the authors themselves [181]. A systemic review and meta-analysis by Hirano et al. in 2019, including data from TRICC, TRISS, and TRICOP, showed no difference in mortality between restrictive and liberal transfusion strategies [182]. The 2021 SSC guidelines adhered to the 2016 strong recommendation favoring a restrictive transfusion strategy with a change in the quality of evidence from strong to moderate [6]. We did not find any major relevant RCT after 2021 addressing this issue. Therefore, in 2023, we believe a restrictive transfusion strategy (<7 g/dL) should be followed for almost all patients with septic shock while acknowledging that a slightly higher threshold (<8 g/dL) may benefit a small subset of patients.

### 5.7. Glucose Control

The 2004 SSC guidelines included a grade D recommendation to maintain blood glucose < 150 mg/dL in patients with severe sepsis [183]. This recommendation was primarily based on a large single-center trial in surgical ICU patients showing a survival benefit with intensive insulin therapy (blood glucose maintained at 80–110 mg/dL) compared to conventional insulin therapy (blood glucose maintained at <215 mg/dL), despite a higher risk of hypoglycemia in the intensive insulin group [184]. In another trial published in 2006 by the same authors, unlike in the surgical ICU, intensive insulin therapy in the medical ICU did not lead to a survival benefit despite reducing morbidity [185]. In 2009, NICE-SUGAR, a large multicenter trial that randomized 6104 medical and surgical patients to either intensive glucose control (blood glucose target 81–108 mg/dL) or conventional glucose control (blood glucose target < 180 mg/dL), showed that intensive glucose control increased 90-day mortality compared to conventional (27.5% vs. 24.9%). This outcome was similar in surgical and medical patients [186]. The 2016 SSC guidelines recommended a blood sugar target of <180 mg/dL in adult patients with sepsis and septic shock based on the results of NICE-SUGAR and other randomized trials and meta-analyses [33]. In 2021, the SSC recommendation to initiate insulin therapy in this population did not change (>180 mg/dL). However, the typical blood glucose target was suggested to be 144–180 mg/dL (compared to 110–180 mg/dL). This change aligned with the American Diabetes Association guidelines and a large meta-analysis suggesting an increase in the hypoglycemia (110–144 mg/dL) target compared to (144–180 mg/dL) [6,187,188].

### 5.8. The Vitamin C Controversy

In 2017, Marik et al. published a small retrospective before–after clinical study (*n* = 47) showing that treatment with intravenous vitamin C, hydrocortisone, and thiamine was associated with a dramatic absolute risk reduction (>30%) in mortality [189]. A similar size and design study should have been nothing but a hypothesis-generating one. Instead, this study gained significant media and public attention, and the “vitamin C cocktail” was portrayed as the cure for sepsis, with some early adopters in the medical field [190,191,192]. This dramatic survival benefit was not replicable in several multicenter randomized controlled trials. CITRIS-ALI showed lower mortality with vitamin C in patients with sepsis and ARDS, but this finding was only a secondary exploratory outcome [193]. The ACTS trial showed no difference, and VITAMINS showed potential harm with vitamin C [194,195]. The 2021 SSC guidelines issued a weak recommendation against intravenous vitamin C based on low-quality evidence [6]. After these guidelines, two randomized controlled trials were published: VICTAS in 2021, showing no difference in mortality, and LOVIT (largest RCT for vitamin C in sepsis; *n* = 872) in 2022, showing a higher risk of death or persistent organ dysfunction at 28 days in the vitamin C group [196,197]. In 2023, cumulative evidence (moderate quality) supports a lack of benefit for high-dose intravenous vitamin C in patients with septic shock from high-income countries. However, this may not be the case in low-income countries, where studies are still needed, and vitamin C deficiency may be present.

## 6. Conclusions and Future Perspectives

Over the last two decades, the management of sepsis evolved toward less invasive strategies. Early recognition of sepsis and prompt intervention remain the cornerstone of sepsis management, although an early goal-directed therapy targeting mixed venous oxygen > 70% and hematocrit > 30% did not offer a significant benefit [6,33]. Early administration of vasopressors through peripheral lines is now considered safe, and a restrictive fluid strategy is at least non-inferior to a liberal one [6,79]. Many questions regarding sepsis diagnosis and management remain unanswered, and the benefit of a particular intervention likely relies on identifying the proper subset of patients. The role and timing of vasopressin, as well as of angiotensin II, remain somewhat controversial [95,104]. The role of immunotherapeutic agents, except for corticosteroids, remains unclear but may prove beneficial once an individualized approach based on the immunological profile of septic patients is adopted [198]. The effect of intravenous immunoglobulin and blood purification is still debatable, given insufficient cumulative evidence to support routine use [6].

The heterogeneity of sepsis patients is likely responsible for the failure of multiple sepsis therapy trials despite treatment success observed in preclinical trials [199]. A large retrospective analysis using data from >60,000 sepsis patients recently identified 4 clinical phenotypes that correlated with host response patterns and clinical outcomes [200]. The identification of these phenotypes may help in the design of future clinical trials and allow for more precise care. Early identification of infected patients who may develop sepsis is critical to decreasing sepsis-associated mortality [5,6]. In the era of machine learning, automated alerting systems were found to have a beneficial effect outside of the ICU, and a recent prospective multi-site study showed that a Targeted Real-time Early Warning System (TREWS) potentially reduced sepsis-related mortality by 3% [201,202].

During the next decade or two, further studies will likely focus on identifying the subset of patients at a higher risk of progressing to sepsis and septic shock using machine learning and identifying different sepsis endotypes and phenotypes to test novel drugs or retest old agents/interventions that failed to show a benefit in a heterogenous sepsis population.

## Figures and Tables

**Table 1:** Evolution of sepsis definitions. MAP: mean arterial pressure, SOFA: Sequential Organ Failure Assessment.

**1992 SEPSIS—1** Bone et al. [3]	**Sepsis** Systemic inflammatory response to infection	**Severe Sepsis** Sepsis associated with organ dysfunction, hypoperfusion, or hypotension	**Septic Shock** Sepsis-induced hypotension despite adequate fluid resuscitation along with the presence of perfusion abnormalities
**2001 SEPSIS—2** Levy et al. [4]	**Sepsis** A clinical syndrome defined by the presence of both infection and a systemic inflammatory response	**Severe Sepsis** Sepsis associated with organ dysfunction, hypoperfusion, or hypotension	**Septic Shock** State of acute circulatory failure characterized by persistent arterial hypotension unexplained by other causes
**2016 SEPSIS—3** Singer et al. [5]		**Sepsis** A life-threatening organ dysfunction caused by a dysregulated host response to infection (SOFA score of ≥2). In-hospital mortality >10%	**Septic Shock** A subset of sepsis with profound circulatory, cellular, and metabolic abnormalities with higher mortality. Vasopressor requirement (MAP ≥ 65 mmHg) and serum lactate level > 2 mmol/L in the absence of hypovolemia. In-hospital mortality > 40%

## Data Availability

No new data were created or analyzed in this study. Data sharing is not applicable to this article.
